# The Influence of Material and Process Parameters on Pressure Agglomeration and Properties of Pellets Produced from Torrefied Forest Logging Residues

**DOI:** 10.3390/ma19020317

**Published:** 2026-01-13

**Authors:** Arkadiusz Gendek, Monika Aniszewska, Paweł Tylek, Grzegorz Szewczyk, Jozef Krilek, Iveta Čabalová, Jan Malaťák, Jiří Bradna, Katalin Szakálos-Mátyás

**Affiliations:** 1 Department of Biosystems Engineering, Institute of Mechanical Engineering, Warsaw University of Life Sciences, Nowoursynowska 164, 02-787 Warsaw, Poland; monika_aniszewska@sggw.edu.pl; 2 Department of Forest Utilization and Forestry Technology, Faculty of Forestry, University of Agriculture in Krakow, Al. Mickiewicza 21, 31-120 Krakow, Poland; pawel.tylek@urk.edu.pl (P.T.); grzegorz.szewczyk@urk.edu.pl (G.S.); 3 Department of Environmental and Forestry Machinery, Faculty of Technology, Technical University in Zvolen, T. G. Masaryka 24, 960 01 Zvolen, Slovakia; jozef.krilek@tuzvo.sk; 4 Department of Chemistry and Chemical Technologies, Faculty of Wood Sciences and Technology, Technical University in Zvolen, T. G. Masaryka 24, 960 01 Zvolen, Slovakia; iveta.cabalova@tuzvo.sk; 5 Department of Technological Equipment of Buildings, Faculty of Engineering, Czech University of Life Sciences Prague, Kamýcka 129, 165 21 Prague, Czech Republic; malatak@tf.czu.cz (J.M.); bradna@tf.czu.cz (J.B.); 6 Institute of Forest and Natural Resource Management, University of Sopron, Bajcsy-Zsilinszky u. 4, 9400 Sopron, Hungary; szakalosne.matyas.katalin@uni-sopron.hu

**Keywords:** biomass, pellets, torrefaction, compressive strength, elemental composition, calorific value

## Abstract

Pellets produced from raw or torrefied shredded logging residues have been investigated in the study. The research material came from pine and spruce stands in Poland, Slovakia, Czechia and Hungary. Torrefaction temperatures (*T_t_*) of 250, 300, and 400 °C were applied. Before pressure agglomeration, 3% wheat flour was added to the torrefaction material as a binding agent. Pellets with a diameter of 8 mm were produced at constant humidity, compaction pressure (*P*) of 140 or 180 MPa and agglomeration temperature (*T_a_*) of 100, 120 or 140 °C. The produced pellets were assessed for their physicomechanical parameters (density, radial compressive strength, compression ratio, modulus of elasticity), chemical parameters (extractive compounds, cellulose, lignin) and energy parameters (ash content, elemental composition, calorific value). The results were subjected to basic statistical analysis and multi-way ANOVA. The produced pellets varied in physical, mechanical, chemical and energy properties. A significant effect of torrefaction temperature, agglomeration temperature and compaction pressure on the results was observed. In terms of physicomechanical parameters, the best pellets were produced from the raw material, while in terms of energy parameters, those produced from the torrefied material were superior. Pellets of satisfactory quality produced from torrefied logging residues could be obtained at *T_t_* = 250 °C, *T_a_* = 120 °C and *P* = 180 MPa. Pellets with specific density of approximately 1.1 g·cm^−3^, radial compressive strength of 3–3.5 MPa, modulus of elasticity of 60–80 MPa and calorific value of 20.3–23.8 MJ·kg^−1^ were produced in the process.

## 1. Introduction

The depletion of fossil fuel reserves with each year has stimulated the search for new energy solutions. Growing public awareness of environmental protection, coupled with the implementation of relevant regulations, induces a shift towards increasingly environmentally friendly heating fuels. As evidenced by research, the combustion of fossil fuels contributes to approximately 89% of global CO_2_ emissions, while changes in land use, particularly deforestation, account for the remaining 11% [1]. Under the ETS2 system, starting in 2027, fossil fuels will face an additional carbon tax. Hence, biomass, which has considerable technical and development potential, emerges as alternative to coal. Biomass is already the world’s third-largest natural source of energy after coal and oil. However, in terms of physicochemical properties, fresh biomass remains a problematic fuel due to its high moisture content, relatively low calorific value, low bulk density, and irregular particle shape and size [2,3]. The literature describes various biomass processing technologies to overcome these drawbacks and limitations. One of the most popular and best known methods is agglomeration, which involves compressing biomass particles to obtain a product with increased volumetric and energy density as well as a uniform shape (pellets, briquettes), facilitating transportation and storage [4,5]. A more recent method for improving the energy properties of biomass is torrefaction. It involves heating biomass at temperatures between 200 and 350 °C in an inert or oxidative atmosphere. This process eliminates most of the unfavourable characteristics of raw biomass.

The torrefied biomass produced in the process is a hydrophobic material with a uniform structure. It has low moisture content, low bulk density and high calorific value [2,6,7,8,9]. The calorific value of raw biomass is approximately 18–19 MJ·kg^−1^, while following the torrefaction process which removes volatile substances, it increases to 25–30 MJ·kg^−1^ [10,11]. The torrefied product has physicochemical properties similar to low-ranked coals (28–30 MJ·kg^−1^) rather than to biomass. The physical properties of biomass fibres change significantly during torrefaction. Thermal degradation of cell wall polymers (hemicelluloses, cellulose and, to some extent, lignin) transforms biomass into a brittle material with hydrophobic properties [12]. Several studies have demonstrated that torrefaction increases biomass combustion efficiency [13,14].

Some authors recommend integrating various biomass processing technologies to improve its physicomechanical and energy properties. Studies suggest that for a single type of biomass, torrefaction followed by mechanical densification of the torrefied product is the most effective approach to improving both physical and chemical properties [15,16,17]. Another strategy discussed in the literature is co-densification, which involves densifying mixtures of various types of biomass and torrefied products to improve the quality of agglomerates (pellets or briquettes) [18,19].

Torrefied biomass can be agglomerated to produce the so-called black pellets [20,21,22]. Agglomeration increases the energy density of the product by 4–8 times and significantly reduces transportation and storage costs [12,23]. In general, there are two methods to produce pellets from torrefied biomass. The first method involves biomass torrefaction and grinding followed by pressure agglomeration to produce appropriately sized pellets. In the second method, crushed raw biomass is pelletised and then torrefied. The former is the more popular of the two.

The densification of torrefied biomass has been investigated in a number of studies. These have determined that torrefied biomass particles do not bond as effectively as in raw biomass, and the produced pellets have poor storage and transportation properties [10,11,24,25,26,27].

Irrespective of the method, preparing durable and strong pellets requires the optimisation of the process conditions during both torrefaction and pelletisation. Companies involved in torrefaction and pressure agglomeration add various binding agents to the primary material, such as glycerine, paraffin, molasses, lignin, flour or bioplastics, to improve fuel quality. Scientific publications on pellets produced from torrefied biomass have focused primarily on examining various materials from which the product is made, describing the resulting changes in the elemental composition and energy density of the solid end-product [10,26,28,29]. Pellet quality has also been studied for specific input materials with respect to hardness [26], durability [10,24] or water resistance [24,29].

In recent years, the use of torrefied biomass with binder additions for pellet production has been studied more intensely. For example, Sarker et al. [30] investigated the co-densification of canola hull, oat hull and barley straw, with pyrolysis bio-oil used as a binding agent. Torrefaction as post-densification treatment markedly increased the calorific value, energy density and carbon content of pellets compared to raw biomass. Similar investigation was conducted by Guo et al. [31], who densified previously torrefied corn straw with fine coal. This method enhanced the physical properties and energy yield ratio of pellets, and reduced NO_x_ and SO_2_ emissions. The aforementioned authors also concluded that pre-torrefaction of biomass was more efficient in terms of energy consumption and end-product quality [29,30,31]. In addition, torrefaction and densification of wood material, such as pine, spruce or fir, were investigated by Ghiasi et al. [24], Shang et al. [10,11,32], Larsson et al. [27], Peng et al. [25,26] and Stelte et al. [33].

Wood represents a complex heterogeneous colloidal system of substances composed of main components (hemicelluloses, cellulose, lignin) and accompanying substances (extractives). The thermal resistance of the fundamental structural components of wood differs. Wood degradation takes place at a relatively low temperature, around 200 °C [34]. Based on Ragan et al. [35], hemicelluloses are the least resistant to thermal decomposition, breaking down in the temperature range of 170–240 °C. Cellulose is more resistant than hemicelluloses; its decomposition is mild up to 250 °C. An intensive thermal decomposition of cellulose occurs in the temperature range of 250–350 °C. Lignin is the most thermally resistant component of wood, with active decomposition occurring at temperatures between 300–400 °C. According to Kim et al. [36], hemicellulose depolymerisation occurs between 180 and 350 °C, the random cleavage of the glycosidic linkage of cellulose between 275 and 350 °C and the degradation of lignin between 250 and 500 °C. There are only a few studies focused on the influence of torrefaction on lignocellulose material structure. Study by Ong et al. [37] describes the impact of torrefaction on chemical structure of lignocellulose materials based on several characterisation methods. At the end of the torrefaction process, the components of hemicelluloses, lignin, and cellulose are decomposed and a high amount of volatile compounds is emitted. Compared to cellulose and lignin, the solid content from torrefaction of hemicelluloses is usually the lowest [38]. Based on Ru et al. [39], torrefaction contributes to the reduction of hemicellulose content from 22% to 4.6% at 300 °C, while cellulose degrades slowly and the lignin content increases.

The literature on the subject includes numerous studies on the torrefaction of specific plant species [6,12,24,25,26,33,40,41], mostly conducted in an inert atmosphere. This indicates difficulties in pelleting torrefied biomass, obtaining high-quality pellets and achieving acceptable production efficiency. In terms of quality, durability and density, biomass pellets are typically inferior to natural wood ones. There has still been no research integrating torrefaction with the mechanical densification of shredded logging residues of various compositions.

Although a number of authors have studied the torrefaction of biomass and its use for the production of densified fuels (pellets, briquettes), according to the authors’ best knowledge none of these studies have addressed the densification of torrefied material produced from shredded logging residues. In the Visegrád Group (V4) countries, the annual potential of logging residues is measured in millions of cubic metres, which could be used for energy purposes. Therefore, it was decided to evaluate the parameters of the process of pellet production from torrefied logging residues and analyse the physicomechanical, chemical and energy properties of the agglomerated product. It should be noted that there is a difference between using chipped clean roundwood and chipped logging residues for torrefaction and agglomeration.

## 2. Materials and Methods

### 2.1. Research Material

The input material for the study consisted of logging residues from pine stands in Poland and Hungary, as well as spruce stands in Czechia and Slovakia. A detailed description of the research material origin is provided in Table 1 below.

Logging residues, including branches with needles, treetops and bark, were shredded in the forest area using a BRUKS 805 CT mobile chipper (Bruks Siwertell Group, Stockholm, Sweden). In the next stage, woodchips were ground in a laboratory using an LMN-100 knife mill (TESTCHEM, Pszów, Poland) to obtain particles <3 mm. The prepared material was torrefied at three temperatures: 250, 300 and 400 °C (*T_t_*). The torrefied material was subjected to pressure agglomeration in order to produce pellets. However, due to low moisture content (approximately 3–4%), particles did not bind together and the torrefied material could not be formed into pellets. Therefore, it was moisturised to increase its moisture content to 13–15% [24], and wheat flour (3% by mass) was added to it as a binding agent. Moisturising and binder addition were based on the method described by Ghiasi et al. [24]. Ultimately, the moisture content of the torrefied material used in the study averaged 13.8 ± 1%. The torrefied material prepared as described above was pelletised at a laboratory station.

After preliminary densification tests, it was decided that further pressure agglomeration of the torrefied material would be conducted at temperatures of 250 and 300 °C. The material torrefied at *T_t_* = 400 °C failed to agglomerate, as its particles did not bind together. It should be noted that 400 °C is outside the generally accepted range of the torrefaction process.

### 2.2. Particle Size Distribution

Particle size distribution was determined for both the raw and torrefied material in accordance with ISO 17827 [42]. A LAB-11-200/UP shaker (EKO-LAB, Brzesko, Poland) was used, equipped with a set of sieves with square apertures of 4.75, 3.35, 2.36, 1.60, 1.18, 0.85, 0.60, 0.425, 0.30, 0.212, 0.15 and 0.10 mm, and a collecting pan [43,44]. A single sample had a mass of 100 ± 0.5 g, and the sieving time was 600 s (measured with a stopwatch with an accuracy of ±1 s). Three replicates were performed for each mixture. In order to determine the percentage composition, the separated fractions from individual sieves were weighed on a WLC 1/10.X2 electronic balance (RADWAG, Radom, Poland) with an accuracy of 0.01 g.

Based on the particle size distribution by mass (*m_u_*) determined in the experiment, the geometric mean of particle size (*X_g_*) together with the dimensionless standard deviation (*S_g_*) and the dimensional standard deviation (*S_gw_*) were computed. The *x*_50_ value, which is the mesh size through which 50% of the material passes, and the cumulative particle size distribution (*m_uk_*) were approximated by the Rosin-Rammler model and distribution parameters according to the methodology described by Lisowski et al. [43,45].

### 2.3. Moisture Content

Moisture content (MC) of the torrefied material was assayed before agglomeration, and of the produced pellets during a static compression test. The drying and weighing method was used in accordance with the requirements of ISO 18134 [46,47,48]. The torrefied material and pellets were dried in a Heraeus UT 6120 drying oven (Kendro Laboratory Products GmbH, Hanau, Germany) for at least 24 h at a temperature of 105 ± 2 °C. The weight before and after drying was determined on a WPS 600/C laboratory balance (RADWAG, Radom, Poland) with an accuracy of 0.01 g.

Throughout the study, the temperature and relative air humidity in the room were monitored using a FHT 100 m (Geo-FENNEL, Kassel, Germany) at a resolution of 0.01 °C and 0.1% with the maximum permissible error of ±2 °C (±1%) Ambient temperature and humidity were approximately 20 °C and 24%, respectively.

### 2.4. Pellet Production

The agglomeration process was carried out on an Inspekt Table 100 tensile testing machine (Hegewald & Peschke GmbH, Nossen, Germany), equipped with a closed-chamber die with an external heater. A constant die temperature within ±2 °C was maintained using an R-703 temperature controller (CZAKI, Nadarzyn, Poland) with a TP-391 NiCr-NiAl Type K thermocouple (CZAKI, Nadarzyn, Poland).

The biomass was compacted using a piston in a closed chamber with an internal diameter (*d*) of 8 mm. The maximum force (*F*) of 7 kN or 9 kN was applied to the material, which corresponded to compaction pressure (*P*) of 140 MPa or 180 MPa, respectively. The piston feed rate was constant at 100 mm·min^−1^. The LabMaster software v. 4.2.4.12 controlling the machine recorded the force with an accuracy of 0.1 N and the feed rate with an accuracy of 0.001 mm. The compaction of each material was performed at three agglomeration temperatures (*T_a_*): 100, 120 and 140 °C. The adopted ranges of agglomeration pressure and temperature corresponded to the methodology described by Lee and Kim [49]. The chamber was filled with crushed material weighing between 1.05 and 1.06 g. After initiating the test procedure, a piston moving within the die compacted the material until the preset maximum force (*F_max_*) was achieved. Then, the piston was stopped and held in a constant position for 15 s. During this period, the stresses within pellets relaxed as a result of constant deformation. In the next stage of the process, the compaction force was released to *F* = 0 N, the die opened and pellets were extruded. In total, 72 measurement series were performed for all combinations of the torrefied and raw material, agglomeration pressure (two values), torrefaction temperature (two values) and agglomeration temperature (three values)—Table 2. At least 12 pellets were produces in each series.

During agglomeration, the control system measured the bulk volume of the material in the die and the pellet volume at *F_max_*. Taking into account the mass of the filled material, the system computed the bulk density ($\rho_{BD}$) and density of pellets at *F_max_* ($\rho_{p\;Fmax}$). Based on the results, the compression ratio (*CR*, %) was determined. A lower value of the ratio corresponds to higher compression and enhanced susceptibility of the material to agglomeration.

(1) $$CR=\frac{V_{p(Fmax)}}{V_{1}}\cdot 100\%$$

where: $V_{1}$—bulk volume of the material in the sleeve before densification, mm^3^; $V_{p(Fmax)}$—volume of pellets in the sleeve at *F_max_*, mm^3^.

After pellet production, a single pellet was removed from the die and its external dimensions (length—*l_p_* and diameter—*d_p_*) were measured with an accuracy of 0.1 mm using an AOS ABSOLUTE Digimatic 150 mm electronic calliper (Mitutoyo Corp., Kawasaki, Japan). Pellet mass (*m_p_*) was determined with an accuracy of 0.001 g using a WPS 210S moisture analyser (RADWAG, Radom, Poland). The pellet dimensions and mass were measured again before radial compression testing, approximately 5–7 days after production.

### 2.5. Unit Density of Pellets

Based on the piston displacement and position registered by the LabMaster software for the maximum compaction force (*F_ma_*_x_) during the densification process, the unit density of pellets inside the die after compression (*ρ_Fmax_*) was computed. Based on *l_p_*, *d_p_* and *m_p_*, the density of a single pellet (*ρ_p_*_48_) was determined with an accuracy of 0.001 g·cm^−3^ before radial compression testing.

### 2.6. Radial Compressive Strength

As pellets stabilise approximately 48 h after production, radial compression tests were performed about 5–7 days after production. Individual pellets were placed on a flat surface and pressed with a stamp until the force at which the sample fractured was achieved. LabMaster software controlling the testing machine recorded displacement with an accuracy of 0.001 mm and force with an accuracy of 0.5 N. Based on *lp* and *dp*, as well as *F_cmax_* at which failure occurred, the maximum compressive stress ($\sigma_{M}$) was computed [50,51]. In addition, the modulus of elasticity (*E*) and strain ($\varepsilon_{cM}$) at *F_cmax_* were determined according to the formulas and method described by Nurek et al. [52].

### 2.7. Chemical Analyses

Spruce and pine wood samples were milled to a particle size of 200–300 µm using a POLYMIX PX-MFC 90D laboratory mill (Kinematica, Lucerne, Switzerland). The content of extractives (EXT) was determined according to ASTM D1107-21 [53] using a mixture of absolute ethanol for analysis (Merck, Darmstadt, Germany) and toluene for analysis (Merck, Darmstadt, Germany) in a volume ratio of 1.0/0.427. Lignin (LIG) content was determined according to Sluiter et al. [54], and cellulose (CEL) content according to Seifert [55]. All measurements were performed in four replicates per sample. The results are expressed on an oven-dry basis relative to unextracted wood.

### 2.8. Energy Parameters of Pellets

The raw material was ground to a particle size of <1.0 mm using an LNM 100 mill (TESTCHEM, Radlin, Poland). Based on the procedures described in ISO 18134 [46,47,48], ISO 18122 [56], ISO 18125 [57], ISO 16993 [58], and ISO 1928 [59], the following parameters were measured and calculated: ash content, elemental content (*C*, *H*, *N*, and *S*), oxygen content (*O*), gross calorific value (*GCV*), and net calorific value (*NCV*). Measurement equipment from LECO Corporation (Benton Harbor, MI, USA) was used for the tests. Ash content was determined using a TGA701 analyzer; *C*, *H*, *N*, and *S* content was determined using a CHN628+S analyzer; and calorific value was determined using an AC600 calorimeter. The mass of the samples used for calorimetric measurements was 1.0 ± 0.01 g. For comparison purposes, all results were converted to a dry state

Samples were weighed using a WPA 40/160-C/1 laboratory balance (Radwag, Radom, Poland) with an accuracy of 0.001 g.

The net calorific value (*NCV_d_*) of the dry material was calculated using Formula (2):

(2) $${NCV}_{d}={GCV}_{d}-212H_{d}-0.8\left(O_{d}+N_{d}\right)\;,\;\;MJ\cdot {kg}^{-1}$$

where: *H_d_*—hydrogen content, *O_d_*—oxygen content and *N_d_*—nitrogen content on a dry basis in %.

The oxygen content (*O_d_*) on a dry basis was calculated using Formula (3):

(3) $$O_{d}=100-{Ash}_{d}-C_{d}-H_{d}-N_{d}-S_{d}-{Cl}_{d},\;\;\%wt.$$

where: *Ash_d_*—ash; %; *C_d_*—carbon; %; *H_d_*—hydrogen; %; *N_d_*—nitrogen; %; *S_d_*—sulphur, %; *Cl_d_*—chlorine, %.

The sulfur content was <0.02%, which was less than the measurement equipment error. The chlorine content was not determined. Therefore, these elements were not included in the calculations of oxygen content (Formula (3)). The research methods used, along with the measurement accuracies, were described in the authors’ earlier works: Piętka et al. [60], Aniszewska et al. [61,62], and Tamelova et al. [63]. All measurements were performed in at least five replicates.

### 2.9. Statistical Analysis

Statistical analyses were performed using Statistica software v. 13 [64]. Basic statistics, analysis of variance (ANOVA) and *post hoc* tests were conducted at the significance level α = 0.05. Differences between mean values were deemed significant at *p* < 0.05.

## 3. Results

### 3.1. Characteristics of the Material

Values of the parameters characteristic of the particle size of the material used to produce pellets are presented in Table 3 below.

The average particle size in samples produced from spruce logging residues varied (*p* < 0.05) depending on the origin of the material (SK, CZ), whereas the torrefaction process itself or torrefaction temperature did not affect particle size (*p* > 0.05). In the case of samples produced from pine logging residues, both the origin (PL, HU) and torrefaction temperature (250, 300 °C) had a statistically significant effect on the particle size of the crushed material (*p* < 0.05), although no significant difference (*p* > 0.05) in the average particle size (*X_g_*) was observed for PW-PL-T300 and PW-HU-T250 materials. For pine wood, the torrefaction process significantly affected particle size (*p* < 0.05) compared to the original raw material.

**Figure 1:**
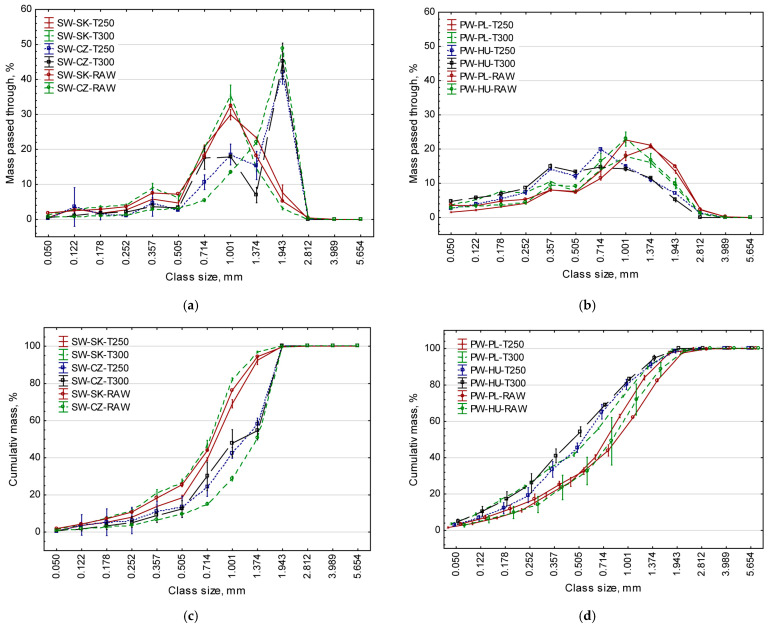
Distribution (**a**,**b**) and cumulative share (**c**,**d**) by mass of particle size fractions of the raw and torrefied material at various temperatures.

The distribution and cumulative share of particle size fractions by mass for all research materials are shown in Figure 1 below.

The particle size distribution differed significantly depending on the material origin (cf. Figure 1a,b). In both cases, a left-skewed pattern can be observed, but pine wood (PW) particles have a flattened and more uniform distribution. This is similar to other types of comminuted biomass described by Lisowski et al. [51], and differs from biomass containing long-fibre particles. In the case of the cumulative share (cf. Figure 1c,d), particle distributions are S-shaped, which is consistent with the information reported in the literature. In all cases, cumulative distributions fitted the Rosin-Ramler model very well (*R*^2^ = 0.83–0.99). Although shredding was performed on the same machine, the aforementioned distributions and particle shape varied depending on species, degree of processing, moisture content and a number of other material and technological parameters. Therefore, the input materials cannot be directly compared with one another in further analyses.

### 3.2. Physicomechanical Parameters

Basic statistics regarding physicomechanical properties of the produced pellets, including mean values and standard deviations for all research combinations, are presented at the end of the article in appendices Table A1 and Table A2.

**Figure 2:**
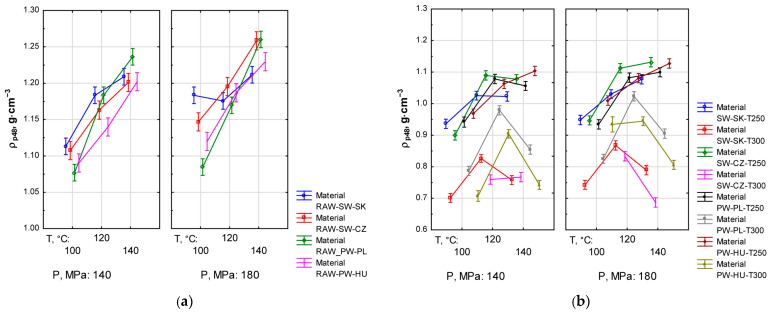
Dependence of pellet density ($\rho_{p48}$) on agglomeration temperature (*T_a_*), torrefaction temperature (*T_t_*) and compaction pressure (*P*) for pellets produced from raw material (**a**) and torrefied material (**b**).

The results for pellet density ($\rho_{p48}$) were compared in two agglomeration pressure groups: *P* = 140 MPa and *P* = 180 MPa (cf. Figure 2). The values determined for the pellets produced from the raw material (*F*_(6, 240)_ = 8.9598, *p* < 0.05) and the torrefied material (*F*_(13, 536)_ = 19.046, *p* < 0.05) indicate significant differences between the analysed groups.

For both the raw and torrefied material, raising the agglomeration pressure from 140 MPa to 180 MPa increased the unit density of pellets. Depending on the test combination, $\rho_{p48}$ increased by approximately 0.05–0.1 g·cm^−3^ for the torrefied material and by approximately 0.03–0.05 g·cm^−3^ for the raw material. These differences were statistically significant (*p* < 0.05), which confirms the effect of pressure on biomass densification.

For pellets produced from the raw material, an upward trend in $\rho_{p48}$ with growing *T_a_* was observed in all cases except RAW-SW-SK at *P* = 180 MPa. For pellets from the torrefied material, the dependence of $\rho_{p48}$ on *T_a_* was nonlinear: raising *T_a_* from 100 to 120 °C caused an increase in $\rho_{p48}$, while a further increase in *T_a_* to 140 °C resulted in a decrease in density, which is particularly visible for pellets made of the torrefied material at *T_t_* = 300 °C. The decrease in $\rho_{p48}$ was not observed for pellets compacted at *P* = 180 MPa and made of the material torrefied at *T_t_* = 250 °C as well as for PW-HU-T250 pellets compacted at *P* = 140 MPa.

The highest acceptable values of $\rho_{p48}$ > 1.0 g·cm^−3^ were recorded for pellets produced from the material torrefied at *T_t_* = 250 °C and agglomerated at 120 or 140 °C and pressure of 140 or 180 MPa, whereas for pellets produced from the raw material this condition was satisfied in all cases.

**Figure 3:**
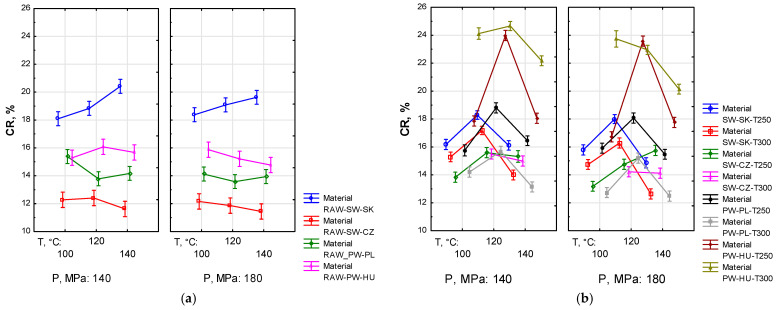
Dependence of compression ratio (*CR*) on agglomeration temperature (*T_a_*), torrefaction temperature (*T_t_*) and compaction pressure (*P*) for pellets produced from raw material (**a**) and torrefied material (**b**).

The statistics regarding the compression ratio (*CR*) for agglomeration pressure groups for pellets produced from the raw material (*F*_(6, 240)_ = 3.2014, *p* < 0.05) or the torrefied material (*F*_(13, 558)_ = 4.9914, *p* < 0.05) indicate significant differences between the analysed groups (cf. Figure 3).

In the case of the raw material, a general downward or stable trend in *CR* with growing *T_a_* was observed, indicating enhanced densification as *T_a_* increased. The exception was RAW-SW-SK, for which the opposite trend occurred. The value of *CR* was also influenced by the type and origin of the material. For the torrefied material, in most cases *CR* increased with an increase in *T_a_* from 100 to 120 °C, then decreased at 140 °C. This implies that compression is the lowest at *T_a_* = 120 °C (with the highest *CR*) while higher at 100 or 140 °C (with lower *CR*). Raising pressure from 140 to 180 MPa typically reduced *CR*; that is, it enhanced compression. Finally, *T_t_* had a positive effect on the material compression. The lowest values of CR, that is, the highest compression, were achieved in three cases at *T_t_* = 300 °C and in one at *T_t_* = 250 °C. For pellets made from the torrefied material, *T_a_* = 120 °C seems to be the transition point at which CR is the highest, that is, where compression is the lowest.

**Figure 4:**
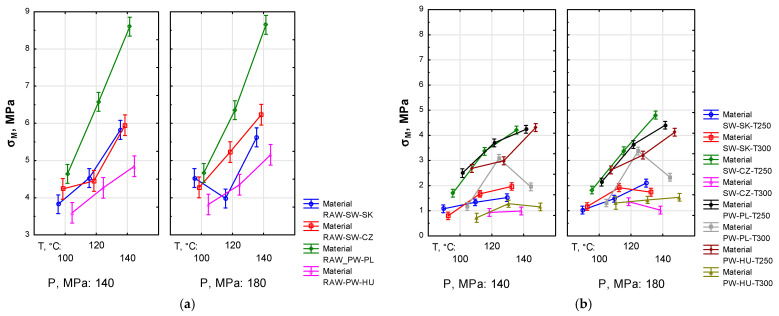
Dependence of compressive stress ($\sigma_{M}$) on agglomeration temperature (*T_a_*), torrefaction temperature (*T_t_*) and compaction pressure (*P*) for pellets produced from raw material (**a**) and torrefied material (**b**).

The statistics regarding the maximum compressive stress ($\sigma_{M}$) for agglomeration pressure groups for pellets produced from the raw material (*F*_(6, 240)_ = 5.0309, *p* < 0.05) or the torrefied material (*F*_(13, 570)_ = 5.849, *p* < 0.05) indicate significant differences between the analysed groups (cf. Figure 4).

For pellets produced from the raw material, the average value of $\sigma_{M}$ ranged from approximately 3.5 MPa to approximately 8.5 MPa, and was higher than for pellets produced from the torrefied material (approximately 0.8–4.8 MPa), which may be attributed to a different structure of samples (degradation due to thermal treatment). There was a marked influence of *T_a_* on $\sigma_{M}$: stress increased linearly with temperature. The exception was RAW-SW-SK at *P* = 180 MPa. Similarly to RAW pellets, for pellets produced from the torrefied material, in most cases an increase in *T_a_* caused an increase in $\sigma_{M}$. However, a different trend was observed for PW-PL-T300, for which $\sigma_{M}$ increased with an increase in *T_a_* from 100 to 120 °C, then decreased at 140 °C. The compaction pressure *P* = 180 MPa generally provided higher $\sigma_{M}$ compared to *P* = 140 MPa, which may suggest that higher agglomeration pressure enhances the compaction and quality of pellets. Finally, lower *T_t_* = 250 °C provided pellets with higher mechanical strength in radial compression.

**Figure 5:**
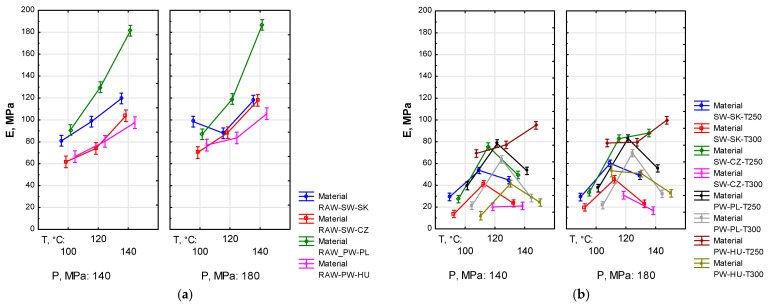
Dependence of modulus of elasticity (*E*) on agglomeration temperature (*T_a_*), torrefaction temperature (*T_t_*) and compaction pressure (*P*) for pellets produced from raw material (**a**) and torrefied material (**b**).

The statistics regarding the modulus of elasticity (*E*) groups for pellets produced from the raw material (*F*_(6, 240)_ = 3.2014, *p* < 0.05) or the torrefied material (*F*_(13, 558)_ = 4.9914, *p* < 0.05), presented in Figure 5 below, indicate significant differences between the analysed groups.

Pellets made from the raw material demonstrated significantly higher stiffness, with *E* ranging from approximately 61 MPa to approximately 186 MPa (cf. Figure 5a). In the case of pellets produced from the torrefied material, the minimum and maximum values of *E* were approximately 12 MPa and slightly below 100 MPa, respectively (cf. Figure 5b). Statistical analysis revealed a marked correlation between an increase in *E* and growing *T_a_*, especially for RAW pellets. A similar relationship was observed with an increase in *P*, but the effect on *E* was lower. A different trend in *E* was observed in pellets produced from the torrefied material, namely a significant increase in *E* with an increase in *T_a_* from 100 to 120 °C, followed by a decrease or stabilisation of *E* with a further increase in *T_a_* to 140 °C. The analysis conducted for most materials also revealed a small but marked increase in *E* with rising *T_a_*. Finally, there was a visible effect of *T_t_*, with *E* being higher for *T_t_* = 250 °C.

### 3.3. Chemical Analysis

After the torrefaction of spruce and pine wood, chemical analyses were carried out, and the obtained results were compared with the original wood. Based on the achieved results (Table 4), it can be stated that different chemical compositions were found in the original samples of spruce and pine wood. The amount of extractives (EXT) in spruce wood was 10.99% (SK) or 2.07% (CZ), while in pine wood it was 7.52% (PL) or 5.23% (HU). The differences in the measurements may be related to the composition of the sample material. Since the material consisted of forest residues, samples contained wood, branches and needles, with the chemical composition of various tree parts being different.

From the results of the chemical analyses (Table 1), an increase in the content of lignin and cellulose with rising torrefaction temperature is evident. Based on the results in Table 1, the share of lignin increased in the range from 65% (pine wood—PL) to 73% (pine wood—HU). The differences can be attributed to the composition of the sample material (wood, bark, needles). Cellulose content increases with growing thermal degradation, which is, however, a result of the method of its determination.

The content of cellulose increased with temperature by 40% (CZ) or 69% (SK) in spruce wood, and by 64% (HU) or 67% (PL) in pine wood. The differences in spruce wood (SK versus CZ) can be attributed to differences in the chemical composition of the original samples. The spruce wood from CZ contained 27% more CEL and 81% less EXT, and CEL is much more resistant to thermal treatment compared to EXT.

### 3.4. Energy Parameters

The energy parameters of the tested raw and torrefied material used to produce pellets, depending on its origin and torrefaction temperature, are presented in Table 5 below.

## 4. Discussion

Most studies on torrefaction and pressure agglomeration use structurally homogeneous material or various mixtures thereof. Forest logging residues are a byproduct of timber harvesting and contain branches, bark, leaves, needles, and different mineral impurities. Both logging residues and the chips produced from them have a heterogeneous structure, with a higher proportion of bark and woven parenchyma compared to roundwood, as well as a higher ash and mineral content, and greater porosity and irregularity of the particles after fragmentation. All these differences directly impact both the torrefaction process and the pressure agglomeration process.

Several authors, particularly Lisowski et al. [45,51,65], Rudolfson et al. [66,67] and Shang et al. [10], have demonstrated that parameters such as particle size, torrefaction rate, moisture content, compaction pressure and agglomeration temperature have a significant influence on the physical and mechanical properties of pellets.

Based on the results obtained, it could be noted that in most combinations, an increase in torrefaction temperature (*T_t_*) resulted in a reduction of the average particle size (*X_g_*), likely to affect the density of the produced pellets. These results confirmed the previous findings by Larsson et al. [27]. This change was observed in absolute values, even though it was not always confirmed by statistical analysis (cf. Table 3). Moderate torrefaction tends to generate an additional fraction of fine particles, especially for spruce (cf. Figure 1). This is likely to improve void filling and particle interactions. There is also evidence that torrefied particles have a porous interior and a surface containing lignin derivatives, which may enhance binding during pressure agglomeration. Studies on wood torrefaction have shown the deposition of lignin on the cell surface, which can act as a binding agent, contributing to the formation of permanent bridges and enhanced particle bonding during densification [67,68,69].

Standard technology for enriching biomass with a low natural binder content (in this case, wood logging residues with a high cetyl content) involves adding flour as a binder during the pelleting process. This binder fills the voids between biomass particles and, after thermal activation of starch and proteins, increases mechanical strength and specific density. According to Samuelsson et al. [70] and Lu et al. [71], due to their binding properties, pellets with added flour are less susceptible to swelling in the presence of moisture and retain their shape better during transport. Higher internal cohesion of the pellets translates directly into less dust (fine fraction) generated during logistics processes. Furthermore, flour acts as a lubricant within the matrix, reducing specific energy consumption during pressing.

One of the key parameters in assessing pellet quality is density, which directly affects transportation, as well as the amount of energy per unit volume. It is assumed that pellets should have specific density >1.0 g·cm^−3^ and bulk density (*BD*) >600 kg·m^−3^ [72,73]. In the conducted study, due to minuscule pellet production at a measurement station, it was not possible to determine bulk density. For pellets produced from the raw material, $\rho_{p48}$ ranged 1.07–1.60 g·cm^−3^, which should be considered a fair result, comparable to that for pellets produced from *Pseudotsuga menziesii* (1.16 g·cm^−3^) [24], conifer cones (1.02 g·cm^−3^) [74], or spruce and beech (1.1–1.4 g·cm^−3^) [75]. Values of $\rho_{p48}$ > 1.0 g·cm^−3^ were also recorded for pellets produced from the material torrefied at *T_t_* = 250 °C and condensed at *T_a_* ≥ 120 °C. These results are similar to those obtained by Ghiasi et al. [24] (1.03–1.20 g·cm^−3^), who produced pellets from torrefied and comminuted *Pseudotsuga menziesii* chips. According to Rudolfson et al. [66,67], torrefaction temperature directly affects pellet density. This was confirmed by the results of the study, as at *T_t_* = 300 °C, in all cases, $\rho_{p48}$ was less than 1.0 g·cm^−3^, ranging 0.68–0.98 g·cm^−3^ (with higher values obtained for *T_a_* = 120 °C). Due to low density, such pellets are not suitable for commercial applications, as according to Stelte et al. [75] their density should be 1.4–1.6 g·cm^−3^. The negative impact of torrefaction temperature on pellet density was described by Sarker et al. [30], who demonstrated increased porosity and relaxation of pellets, indicating that a more porous internal microstructure and increased brittleness have an unfavourable effect on the densification process. Consequently, in some research materials (i.e., SW-CZ-T300), particles did not bind and pellets could not be produced.

Furthermore, the conducted study has confirmed that higher agglomeration pressure contributes to enhanced densification and reduces the number of pores in the pellet structure. Stelte et al. [75] also demonstrated this relationship and an increase in wood pellet density from approximately 0.8 g·cm^−3^ at *P* = 50 MPa to approximately 1.4 g·cm^−3^ at *P* = 600 MPa. This was also confirmed by Lee and Kim [49], who obtained pellets with density of 1.1–1.2 g·cm^−3^ for *Populus*, *Quercus*, *Robinia* and *Pinus* species at agglomeration pressure of 100–200 MPa and agglomeration temperature of 100–130 °C, which corresponds to the results obtained in this study. In addition to pressure, an increase in *T_a_* also increases pellet density and enhances biomass particle binding in the raw material—partly owing to the plasticisation of natural binders, such as lignin, hemicelluloses and thermal degradation products. This also applies to pellets produced from the torrefied material at *T_t_* = 250 °C.

The trend in $\rho_{p48}$ shown in Figure 2b above could be explained by physicochemical processes occurring in biomass of different composition and origin. At a temperature of approximately 120 °C, lignin plasticises, which enhances particle binding and reduces voids. At a high temperature *T_a_* = 140 °C, partial drying and stiffening of particles may occur, hindering their permanent binding and potentially leading to lower pellet density. Both the type of biomass and *T_t_* influenced the absolute density values and the mean values, which differed significantly (*p* = 0.05). However, it does not undermine the general relationship, as all materials reacted similarly.

A parameter indicating the susceptibility to compaction is the compression ratio (*CR*), determined for the particular material inside the compaction sleeve. As reported by Lee and Kim [49], *CR* is typically lower for hardwood than softwood. However, this study investigated only logging residues of softwood species (cf. Figure 3). For most RAW materials, a significant downward trend in *CR* with growing *T_a_* was observed, which indicates greater compaction. In one case (RAW-SW-SK), the trend was opposite. The differences are consistent with the conclusions of Holm et al. [76], and may be attributed to different material composition, structure and chemical composition. *CR* characteristics of the torrefied materials were utterly different. The highest compression was achieved at *T_t_* = 100 and 140 °C. In this case, the degree of compression may depend on the hardness of particles [76]. The harder the particles, the lower their compression. This means that softer materials (RAW) are more susceptible to compression than harder ones (torrefied). Statistical analysis also revealed significant differences in mean values (*p* < 0.05) with respect to both torrefaction and agglomeration pressure and temperature. In general, higher material compression should correspond to higher pellet density and mechanical strength [49]. While this has been confirmed for the RAW material, this relationship was not observed for the torrefied material.

Radial compressive strength is a parameter used in assessing pellet mechanical durability during transportation and storage. Pellets with higher strength are less susceptible to cracking, which ensures better integrity throughout the supply chain and higher efficiency in practical applications. According to Brunerova [77], compressive strength of good quality pellets should exceed 20 MPa; however, her research was based on pellets produced from poultry farm litter. According to Lisowski et al. [78] and Nguyen et al. [79], $\sigma_{M}$ > 1.0 MPa is sufficient to ensure good quality of pellets.

As reported by Dyjakon et al. [80], torrefaction causes a significant decrease in the mechanical strength of pellets produced from the torrefied material compared to those made from the raw material, which was also confirmed by the authors of this publication. Dyjakon et al. [80] conducted their research, among others, for pellets produced from pine wood chips at *T_t_* = 200 or 300 °C. They indicated that a decrease in strength is related to the decomposition of lignocellulosic compounds. However, they did not observe an effect of *T_t_* on the mechanical strength of pellets.

In pellets produced from the raw material, there was a clear dependence of $\sigma_{M}$ on *T_a_* and *T_t_*, and statistical analysis revealed significant differences in mean values of $\sigma_{M}$ with changes in *T_t_*, *T_a_* and *P*. The highest $\sigma_{M}$ was recorded for *T_a_* = 140 °C. Assuming, after Lisowski et al. [78] and Nguyen et al. [79], the minimum compressive stress at 1.0 MPa, most pellets produced from the torrefied material may be deemed to meet this condition (cf. Figure 4). However, for highly brittle pellets obtained from the torrefied material, $\sigma_{M}$ > 2.0 MPa should be required as a minimum. In this study, this condition was met by pellets produced from the material torrefied at *T_t_* = 250 °C. This is confirmed by the study by Stelte et al. [33], who compressed pellets made from spruce wood torrefied at *T_t_* = 250 °C and obtained $\sigma_{M}$ close to or exceeding 2.0 MPa. They also found that increasing *T_t_* above 250 °C led to a reduction in compressive stress, which was also confirmed in this study; moreover, at certain values it was not possible to produce pellets.

Modulus of elasticity (*E*) indicates the susceptibility of pellets to deformation under the action of force. Statistical analysis revealed significant differences in the mean values of *E* (*p* < 0.05), which implies that *T_t_*, *T_a_* and *P* influence the results. In the case of pellets produced from the raw material, there was a significant correlation, as *E* increased with growing *T_a_* (cf. Figure 5). In pellets produced from the torrefied material, a significant increase in *E* was observed with an increase in *T_a_* from 100 to 120 °C. Despite the expected further increase at *T_a_* = 140 °C, an actual decrease in *E* occurred. As reported by Wilczyński and Gogolin [81], changes in the elasticity of plant-derived materials are largely dependent on their moisture and density, while according to Bashaiwoldu et al. [82], elasticity of pellets also depends on their porosity. According to the adopted methodology, pellets were stored for several days after production to enable water loss and bond stabilisation. Water loss increases hardness and strengthens intermolecular bonds, raising the compressive strength of pellets. The observed changes in *E* can be attributed to the condition and chemical composition of materials.

The conducted study has indicated that changes in the density and radial compressive strength of pellets are related to the condition of the material, torrefaction temperature and agglomeration temperature. Pellets made from the raw material were characterised by high density and strength, which implies the importance of torrefaction temperature as well as cellulose, hemicelluloses and lignin content. The temperatures of thermal decomposition of hemicelluloses and cellulose are in the range of 220–315 °C and 315–400 °C, respectively. Torrefaction is carried out at temperatures of 200–300 °C [83]. Based on the research by Chen and Kuo [84], it has been established that temperature has a significant impact on torrefaction results. According to torrefaction temperature, the torrefaction process can be categorised into light torrefaction (200–235 °C), mild torrefaction (235–275 °C) and severe torrefaction (275–300 °C). During light torrefaction, hemicelluloses undergo some thermal degradation, while cellulose and lignin undergo slight or almost no degradation [85], whereas in heavy torrefaction, cellulose undergoes extensive thermal degradation. Lignin is the most difficult to degrade thermally, and its mass loss over the aforementioned temperature range is low. Its mass loss after torrefaction at lower temperatures is not prominent, and its energy density or calorific value increases only slightly. During torrefaction at higher temperatures, cellulose and hemicellulose fibers are largely dissolved. Because hemicelluloses and cellulose are the main components of biomass, significant removal thereof results in a considerable reduction in mass and energy yield, even though fuel energy density increases markedly. This is related to lignin left in biomass, which does not decompose easily.

Torrefaction at 400 °C is problematic from a thermodynamic and process point of view, as it exceeds the standard range for torrefaction (usually 200–350 °C) and enters the pyrolysis regime in the case of woody biomass [86] and lignocellulosic feedstocks [87]. The inconsistency and failure of agglomeration at this temperature can be attributed to chemical and energetic limitations. While standard torrefaction targets hemicellulose, at 400 °C, there is a massive decomposition of both cellulose and lignin. Lignin functions as a natural thermoplastic binder in biomass. Its excessive degradation at 400 °C leads to the breakdown of the internal structure (agglomeration fails) because there is no polymer matrix capable of holding the particles together [88]. Release of volatile substances: At 400 °C, the production of tars and gases increases dramatically at the expense of the solid residue (biochar) [89]. This mass loss creates pores and brittleness in the material, which makes stable agglomeration or pelletization impossible. At temperatures above 300–350 °C, the mass yield drops sharply, which worsens the overall energy balance of the process [90]. The energy consumed for heating to 400 °C and the loss of chemical energy in the released gases make the process for producing solid fuel inefficient. The agglomeration mechanism requires a certain amount of moisture or the presence of low-molecular-weight substances in a plastic state. At 400 °C, the material is too dry and “overburned” (char), which leads to high abrasiveness and low mechanical resistance of the resulting product.

This study has clearly demonstrated correlations between the density, radial compressive strength and modulus of elasticity of pellets (cf. Figure 2, Figure 4 and Figure 5). Such patterns are evident for pellets produced from the raw material and, to a lesser extent, for pellets from the torrefied material. All these parameters have shown marked dependence primarily on torrefaction temperature and, slightly less, on compaction pressure. There are also notable differences depending on the type of the research material.

Furthermore, the study results have clearly indicated that there are specific types of materials (origin, torrefaction) and densification process parameters (pressure, temperature) that make it possible to obtain pellets with satisfactory density and compressive strength. It has also been observed that as *T_a_* increased, the lateral surface of pellets became more smooth and glassy. This is consistent with previous observations by other researchers [91,92].

Treetops, branches and needles contain a higher proportion of extractives and a lower proportion of lignin (LIG) and cellulose (CEL) compared to the wood part of the trunk [93]. According to study results, the yield of extractives decreased significantly. Some authors report an increase in extractive yield during thermal treatment of wood, mainly due to the products of the thermal decomposition of lignin macromolecules [94]. Charring of wood caused a reduction in extractives due to their lower solubility in organic solvents, which is caused by condensation reactions. A decrease in extractives in the charred layer was also observed under other various thermal loading conditions [95,96].

The increase in lignin content is consistent with the generally accepted phenomenon that during thermal treatment of wood, its proportion increases due to its greater thermal stability compared to carbohydrates, as well as due to its condensation [69]. An increase in lignin content due to thermal effects has been observed in various heating methods (Windeisen et al. [97]), with structural differences in lignin also being apparent. In addition to condensation reactions, lignin also undergoes degradation, even under milder conditions [98]. Nanou et al. [99] reported intense structural transformations of lignin during thermal processing of wood, involving cleavage of β-O-4 bonds and strong recondensation reactions.

According to Kučerová et al. [100], during thermal treatment, cellulose undergoes charring and cross-linking, which increases its yield in gravimetric determination, and the total analysis of wood significantly exceeds the value of 100%. Similar results in the analysis of thermally degraded spruce wood by radiant heating at a heat flux of 50.5 kW·m^−2^ were also achieved in the study by Čabalová et al. [101]. The authors reported an increase in CEL by 56% and LIG by 71% compared to the original wood.

As the suitability of torrefied pellets for subsequent applications is also assessed by their energy properties, their elemental composition and calorific value have also been investigated. As expected, the elemental analysis has shown an increase in carbon content and a reduction in oxygen content with growing torrefaction temperature, and, consequently, a marked increase in the C/O ratio. The higher carbon-to-oxygen ratio was also consistent with the increased calorific value of the torrefied biomass compared to the RAW material (cf. Table 5). Changes in the elemental composition also included a decrease in hydrogen content with growing carbon content. According to the study by Ghiasi et al. [24], this can be attributed to an increase in the hydrophobicity of pellets.

A rise in calorific value is a typical effect of mild to severe torrefaction. The increase is particularly rapid within the temperature range of 250–300 °C. Torrefied biomass acquires properties similar to fossil fuels (mainly coal), which positively affects combustion efficiency. Studies have shown that an increase in carbon content coupled with loss of oxygen and hydrogen with growing *T_t_* increases calorific value because the specific energy of the material becomes more concentrated per unit volume. Higher torrefaction temperatures lead to the removal of the volatile fraction and mass concentration, which increases both ash and nitrogen content [24]. The calorific value of pellets produced from the material torrefied at *T_t_* = 250 or 300 °C (cf. Table 5), determined in the study, corresponds to the values reported by Stelte et al. and Bilgin et al. (20–27 MJ·kg^−1^) [40,41]. According to the study results (cf. Table 5), although figures for different combinations vary, the trends seem consistent. Torrefaction had a similar effect on all samples, and the observed variations are most likely attributable to differences in the chemical structure of the raw material (lignin/cellulose/hemicelluloses content).

While the energy density increases with increasing temperature during torrefaction, the total energy yield decreases sharply, as seen with lignocellulosic feedstocks [102]. At temperatures above 300 °C (and even higher at 400 °C), the energy losses in the off-gas exceed the energy gain in the solid biomass [90]. If the process requires more external energy for heating than the increase in the calorific value of the final product, the process loses its “enhancement” status. It becomes an inefficient thermal destruction [103]. Pelletization of torrefaction at 400 °C requires up to 50–100% more mechanical energy than for raw biomass due to the loss of natural lubricants (resins) [104]. Failure of natural agglomeration (due to lignin degradation) forces the addition of external binders, as recommended by Butler et al. [105] in the form of solid lignin by-product obtained from pulp and paper processing, biomass tar obtained from biomass pyrolysis, high oil pitch and lime, which increases operating costs and can negatively affect the emission profile during combustion [106].

Currently, the applications of torrefied products are significantly expanding beyond traditional co-combustion in the energy sector [107]. Given the technological limits and pressure for decarbonization, they are gaining ground especially in high-emission industries and in the circular economy [108]. From 2025, the use of torrefied biomass as a replacement for fossil coal and coke in the steel industry, as well as reducing agents for the reduction of iron oxides to steel, is accelerating [109].

The advantage of using torrefied coal (TC) is that its use as an adsorber for wastewater treatment is proving to be a more effective adsorbent than classic biochar from high-temperature pyrolysis, as shown by the research of Suriyakumar et al. [110]. It is used to immobilize and filter pollutants in industrial wastewater. Torrefied biomass serves as an advanced material for the production of sustainable carbon materials with high added value, such as energy storage, plastics and composites, or a replacement for technical carbon black in the production of dyes and rubber compounds [103].

In agriculture, emphasis is placed on integrating torrefaction into sustainable agricultural systems for soil improvement and long-term carbon sequestration [111,112].

Based on the study results regarding the chemical, physicomechanical and energy parameters, it can be assumed that it is possible to use the technology of agglomeration of the torrefied biomass derived from comminuted forest logging residues, the composition of which is not homogeneous. However, this seems justified if the ultimate fuel to be burned in boilers would be pellets. Then, high density, mechanical durability and good energy parameters of pellets would be required to achieve high efficiency of pellet feeding and combustion.

## 5. Conclusions

Attempts to improve the physical and energy parameters of plant biomass through pelletisation have stimulated market growth and the search for new developments in this area. Fuel in the form of pellets makes transportation and storage easier and more economical, and facilitates automated feeding.

The hydrophobic properties and lower calorific value of raw biomass have prompted the search for new solutions to improve these parameters. Torrefaction is one of such solutions, as it improves energy properties and increases the resistance of fuel to moisture absorption, making it less susceptible to bacteria that can degrade it. However, torrefaction can negatively impact the pelleting process and, ultimately, the physicomechanical properties of produced pellets.

This study has demonstrated that it is possible to produce pellets from shredded and torrefied logging residues, and thus obtain a material that could be used for energy purposes. Although pellets produced from the torrefied material have lower density and lower compressive strength compared to those made from the raw material, they have a significantly higher calorific value, which contributes to the accumulation of energy per unit volume. Furthermore, combustion of the former reduces the emissions of harmful gases to the environment, a factor of paramount importance nowadays.

Based on this study and its results, a technological line for the production of pellets from torrefied logging residues could be designed, taking into account the process parameters that would yield the best pellets in terms of specific density, mechanical compressive strength and energy parameters. However, it should be noted that torrefaction requires additional energy inputs in the energy balance, which calls for further research on the economic viability of the process.

In view of the study results, it may be concluded that, within the investigated range of process parameters, pellets of satisfactory quality produced from torrefied logging residues can be obtained at torrefaction temperature of 250 °C, agglomeration temperature of 120 °C and compaction pressure of 180 MPa. In the process, pellets with specific density of approximately 1.1 g cm^−3^, radial compressive strength of 3–3.5 MPa, modulus of elasticity of 60–80 MPa and calorific value of 20.3–23.8 MJ·kg^−1^ are obtained.

Pellets produced from the raw material demonstrate superior physicomechanical properties. At agglomeration pressure of 140 MPa or 180 MPa, regardless of agglomeration temperature, pellets with density of 1.1–1.26 g·cm^−3^, radial compressive strength of 4–8.5 MPa and elastic modulus of 60–180 MPa can be obtained. However, pellets from fresh logging residues have lower calorific value, ranging from 17.4–19.2 MJ·kg^−1^, i.e., 15–20% lower than pellets made from torrefied wood at *T_t_* = 250 °C. In terms of energy properties, increasing the *T_t_* to 300 °C is advantageous, resulting in an increase in *NCV* of more than 30% compared to the raw material; however, pellets of satisfactory quality cannot be obtained.

The study results indicate the need for further research, particularly determining the net energy yield, and establishing whether the costs of torrefaction will be offset by energy gains from the higher calorific value of pellets produced from the torrefied material.

## Figures and Tables

**Table 1:** Type and origin of the research material.

Country	Species	Type of Operation	Stand Characteristics
Poland (PL)	Pine wood (PW)	Clear-cutting	Pine stand, *Pinus sylvestris*, 85 years old, admixture of deciduous shrubs
Hungary (HU)	Pine wood (PW)	Clear-cutting	Pine stand, *Pinus sylvestris* with a small proportion of *Pinus nigra*, 80 years old, admixture of shrubs
Slovakia (SK)	Spruce wood (SW)	Thinning	Spruce stand, *Picea abies*, 40 years old, admixture of beech
Czechia (CZ)	Spruce wood (SW)	Thinning	Spruce stand, *Picea abies*, 68 years old

**Table 2:** Materials and process parameters used in individual test variants.

Materials	*T_t_*	*T_a_*	*P*
	°C		MPa
RAW	250	100	140
SW-SK	300	120	180
SW-CZ	400 *	140	
PW-PL			
PW-HU			

Note: *—the material torrefied at *T_t_* = 400 °C was used only for chemical and energetic analyses; SW—spruce wood; PW—pine wood; SK—Slovakia; CZ—Czechia; PL—Poland; HU—Hungary.

**Table 3:** Values of characteristic parameters of particle size distribution for the raw and torrefied material used for pellet production.

	*X_g_*	*S_g_*	*S_gw_*	*n*	*R* ^2^	*X*_50_
Spruce						
SW-SK-RAW	0.74 ^a^	2.06	2.85	1.51	0.97	0.70
SW-SK-T250	0.83 ^a^	1.91	2.73	1.47	0.83	0.74
SW-SK-T300	0.71 ^a^	1.99	2.53	1.59	0.97	0.67
SW-CZ-RAW	1.29 ^b^	1.86	4.04	1.76	0.95	1.10
SW-CZ-T250	1.12 ^b^	2.01	4.17	1.90	0.92	0.99
SW-CZ-T300	1.12 ^b^	1.95	3.85	1.84	0.94	0.97
Pine						
PW-PL-RAW	0.73 ^d^	2.53	4.53	1.30	0.98	0.65
PW-PL-T250	0.82 ^a^	2.19	3.59	1.50	0.99	0.77
PW-PL-T300	0.58 ^b^	2.54	3.68	1.27	0.99	0.54
PW-HU-RAW	0.70 ^d^	2.30	3.46	1.38	0.98	0.66
PW-HU-T250	0.57 ^b^	2.31	2.85	1.35	0.99	0.57
PW-HU-T300	0.48 ^c^	2.44	2.74	1.26	0.97	0.45

Note: SW—spruce wood; PW—pine wood; SK—Slovakia; CZ—Czechia; PL—Poland; HU—Hungary; T250, T300—torrefaction temperature, °C; RAW—primary material before torrefaction; *X_g_*—geometric mean of milled walnut shell particle size, mm; *S_g_*—dimensionless standard deviation; *S_gw_*—dimensional standard deviation; *n*—constant characteristic of the material, which is a measure of the steepness of the distribution curve; *R*^2^—coefficient of determination; *X*_50_—mesh size through which 50% of the test material by weight passes, mm; ^a, b, c, d^—homogeneous material groups for different wood species for the significance level α = 0.05.

**Table 4:** Amounts of extractives, lignin and cellulose in analysed spruce and pine wood (results are expressed in % oven-dry wood) after torrefaction.

Chemical Composition		Original	Torrefaction Temperature		
			250 °C	300 °C	400 °C
Spruce—SK	EXT	10.99 ± 0.17	6.6 ± 0.02	4.2 ± 0.03	0.24 ± 0.00
	LIG	32.55 ± 0.29	56.6 ± 0.19	91.79 ± 0.11	96.43 ± 0.08
	CEL	31.80 ± 0.15	68.18 ± 0.08	91.77 ± 0.99	103.66 ± 0.45
Spruce—CZ	EXT	2.07 ± 0.08	4.08 ± 0.07	3.97 ± 0.01	0.11 ± 0.00
	LIG	27.49 ± 0.02	36.34 ± 0.08	63.92 ± 0.14	99.63 ± 0.10
	CEL	43.74 ± 0.12	59.75 ± 0.16	81.01 ± 0.22	73.23 ± 0.17
Pine—PL	EXT	7.52 ± 0.22	5.90 ± 0.21	4.42 ± 0.02	0.29 ± 0.00
	LIG	33.03 ± 0.24	48.50 ± 0.13	80.60 ± 0.24	94.05 ± 0.36
	CEL	34.02 ± 0.18	69.49 ± 0.57	83.56 ± 0.11	103.53 ± 0.11
Pine—HU	EXT	5.23 ± 0.08	5.83 ± 0.04	2.46 ± 0.02	0.19 ± 0.00
	LIG	26.16 ± 0.22	55.10 ± 0.18	93.30 ± 0.17	97.02 ± 0.14
	CEL	37.41 ± 0.22	64.48 ± 0.31	94.10 ± 0.32	102.84 ± 0.17

Note: SK—Slovakia; CZ—Czechia; PL—Poland; HU—Hungary; EXT—extractives; LIG—lignin; CEL—cellulose.

**Table 5:** Mean values and standard deviations of ash content, elemental composition, heat of combustion and calorific value, and C/O ratio for different materials and torrefaction temperatures (all figures on a dry basis).

Material	*A_d_*	*C_d_*	*H_d_*	*N_d_*	*O_d_* *	*C/O* *	*GCV_d_*	*NCV_d_* *
			%				MJ·kg^−1^	
Spruce								
SW-CZ-RAW	0.39 ^a^ (±0.03)	51.40 ^a^ (±0.16)	5.98 ^a^ (±0.02)	0.09 ^a^ (±0.02)	42.14	1.22	20.18 ^a^ (±0.03)	18.88
SW-CZ-T250	0.42 ^a^ (±0.04)	54.83 ^b^ (±0.26)	5.87 ^b^ (±0.06)	0.11 ^a^ (±0.04)	38.77	1.41	21.64 ^b^ (±0.09)	20.37
SW-CZ-T300	0.58 ^b^ (±0.04)	62.86 ^c^ (±0.22)	5.49 ^c^ (±0.02)	0.14 ^a^ (±0.03)	30.95	2.03	24.46 ^c^ (±0.06)	23.27
SW-CZ-T400	1.06 ^c^ (±0.03)	81.15 ^d^ (±1.97)	3.39 ^d^ (±0.15)	0.25 ^b^ (±0.06)	14.15	5.73	31.48 ^d^ (±0.06)	30.75
SW-SK-RAW	3.04 ^a^ (±0.09)	51.90 ^a^ (±0.16)	5.95 ^a^ (±0.05)	0.43 ^a^ (±0.03)	38.68	1.34	20.50 ^a^ (±0.04)	19.21
SW-SK-T250	3.43 ^b^ (±0.16)	60.76 ^b^ (±0.24)	5.45 ^b^ (±0.03)	0.62 ^b^ (±0.10)	30.13	2.02	24.21 ^b^ (±0.09)	22.92
SW-SK-T300	5.68 ^c^ (±0.17)	75.51 ^c^ (±0.31)	4.42 ^c^ (±0.02)	0.92 ^c^ (±0.03)	16.11	4.67	29.35 ^c^ (±0.31)	28.06
SW-SK-T400	7.80 ^d^ (±0.25)	80.80 ^d^ (±0.59)	2.96 ^d^ (±0.02)	1.00 ^c^ (±0.03)	12.21	6.62	30.14 ^d^ (±0.08)	28.84
Pine								
PW-PL-RAW	4.20 ^a^ (±0.73)	45.78 ^a^ (±0.49)	6.21 ^a^ (±0.31)	0.28 ^a^ (±0.04)	44.70	1.23	18.72 ^a^ (±0.63)	17.43
PW-PL-T250	13.19 ^b^ (±4.21)	56.99 ^b^ (±2.62)	4.86 ^b^ (±0.36)	0.41 ^b^ (±0.05)	34.71	1.64	22.19 ^b^ (±0.08)	20.90
PW-PL-T300	11.93 ^b^ (±3.61)	65.02 ^c^ (±4.51)	4.35 ^c^ (±0.45)	0.52 ^c^ (±0.04)	27.07	2.40	23.86 ^c^ (±0.42)	22.56
PW-PL-T400	22.07 ^c^ (±3.48)	71.90 ^d^ (±5.75)	2.86 ^d^ (±0.29)	0.62 ^d^ (±0.04)	21.58	3.33	25.09 ^d^ (±0.11)	23.80
PW-HU-RAW	2.98 ^a^ (±0.39)	50.27 ^a^ (±0.23)	5.89 ^a^ (±0.02)	0.26 ^a^ (±0.03)	40.53	1.24	19.53 ^a^ (±0.22)	18.23
PW-HU-T250	5.48 ^b^ (±0.50)	60.69 ^b^ (±0.43)	5.23 ^b^ (±0.09)	0.60 ^b^ (±0.07)	30.44	1.99	23.88 ^b^ (±0.02)	22.59
PW-HU-T300	7.37 ^b^ (±0.95)	76.71 ^c^ (±0.83)	3.86 ^c^ (±0.10)	0.72 ^c^ (±0.03)	15.67	4.89	28.95 ^c^ (±0.21)	27.66
PW-HU-T400	9.85 ^c^ (±0.88)	78.53 ^c^ (±1.13)	2.91 ^d^ (±0.06)	0.83 ^d^ (±0.02)	14.68	5.35	29.60 ^d^ (±0.13)	28.30

Note: *—computed values; ^a, b, c, d^—homogeneous groups of means for different material origin groups. Statistically significant differences for *p* < 0.05 at the significance level α = 0.05.

## Data Availability

The original contributions presented in this study are included in the article. Further inquiries can be directed to the corresponding author.

## Appendix A

**Table A1:** Basic statistics (means and standard deviations) regarding the physicomechanical properties of pellets produced from the raw and torrefied material.

Material	*P*	*T_a_*	*ρ_BD_*	*ρ_p(Fmax)_*	*CR*	*ρ_p_*_48_
	MPa	°C	g·cm^−3^	g·cm^−3^	%	g·cm^−3^
SW-SK-T250	140	100	0.295 (±0.008)	1.823 (±0.024)	16.18 (±0.48)	0.936 (±0.029)
SW-SK-T250	140	120	0.311 (±0.009)	1.704 (±0.014)	18.28 (±0.50)	1.025 (±0.023)
SW-SK-T250	140	140	0.307 (±0.010)	1.905 (±0.007)	16.11 (±0.54)	1.023 (±0.022)
SW-SK-T250	180	100	0.298 (±0.006)	1.887 (±0.02)	15.78 (±0.27)	0.948 (±0.031)
SW-SK-T250	180	120	0.313 (±0.007)	1.740 (±0.024)	18.00 (±0.53)	1.030 (±0.032)
SW-SK-T250	180	140	0.298 (±0.010)	2.003 (±0.017)	14.87 (±0.5)	1.077 (±0.016)
SW-SK-T300	140	100	0.226 (±0.008)	1.476 (±0.013)	15.29 (±0.56)	0.702 (±0.038)
SW-SK-T300	140	120	0.244 (±0.011)	1.423 (±0.063)	17.18 (±0.56)	0.826 (±0.043)
SW-SK-T300	140	140	0.208 (±0.005)	1.485 (±0.018)	13.99 (±0.41)	0.758 (±0.033)
SW-SK-T300	180	100	0.234 (±0.006)	1.586 (±0.021)	14.74 (±0.37)	0.743 (±0.032)
SW-SK-T300	180	120	0.245 (±0.006)	1.506 (±0.067)	16.28 (±0.58)	0.868 (±0.032)
SW-SK-T300	180	140	0.202 (±0.005)	1.598 (±0.015)	12.64 (±0.26)	0.789 (±0.027)
SW-CZ-T250	140	100	0.225 (±0.006)	1.624 (±0.010)	13.83 (±0.35)	0.900 (±0.025)
SW-CZ-T250	140	120	0.262 (±0.009)	1.681 (±0.016)	15.59 (±0.50)	1.090 (±0.03)
SW-CZ-T250	140	140	0.210 (±0.009)	1.444 (±0.181)	15.32 (±0.35)	1.078 (±0.019)
SW-CZ-T250	180	100	0.223 (±0.007)	1.691 (±0.006)	13.18 (±0.41)	0.947 (±0.026)
SW-CZ-T250	180	120	0.255 (±0.007)	1.730 (±0.009)	14.76 (±0.36)	1.112 (±0.012)
SW-CZ-T250	180	140	0.220 (±0.009)	1.400 (±0.009)	15.75 (±0.66)	1.132 (±0.007)
SW-CZ-T300	140	100	n.a.	n.a.	n.a.	n.a.
SW-CZ-T300	140	120	0.224 (±0.004)	1.447 (±0.065)	15.49 (±0.87)	0.760 (±0.049)
SW-CZ-T300	140	140	0.222 (±0.004)	1.485 (±0.022)	14.98 (±0.39)	0.767 (±0.024)
SW-CZ-T300	180	100	n.a.	n.a.	n.a.	n.a.
SW-CZ-T300	180	120	0.221 (±0.003)	1.551 (±0.007)	14.22 (±0.24)	0.833 (±0.017)
SW-CZ-T300	180	140	0.204 (±0.006)	1.441 (±0.014)	14.12 (±0.46)	0.687 (±0.039)
PW-PL-T250	140	100	0.258 (±0.013)	1.636 (±0.019)	15.75 (±0.80)	0.942 (±0.037)
PW-PL-T250	140	120	0.308 (±0.012)	1.637 (±0.015)	18.8 (±0.66)	1.078 (±0.018)
PW-PL-T250	140	140	0.273 (±0.035)	1.662 (±0.092)	16.45 (±2.2)	1.056 (±0.028)
PW-PL-T250	180	100	0.274 (±0.010)	1.721 (±0.009)	15.91 (±0.59)	0.934 (±0.031)
PW-PL-T250	180	120	0.304 (±0.010)	1.682 (±0.011)	18.07 (±0.54)	1.082 (±0.011)
PW-PL-T250	180	140	0.277 (±0.013)	1.794 (±0.028)	15.47 (±0.68)	1.100 (±0.013)
PW-PL-T300	140	100	0.228 (±0.013)	1.602 (±0.039)	14.20 (±0.49)	0.787 (±0.040)
PW-PL-T300	140	120	0.257 (±0.007)	1.636 (±0.023)	15.69 (±0.25)	0.979 (±0.022)
PW-PL-T300	140	140	0.211 (±0.007)	1.603 (±0.026)	13.13 (±0.41)	0.854 (±0.026)
PW-PL-T300	180	100	0.214 (±0.007)	1.683 (±0.021)	12.73 (±0.42)	0.825 (±0.019)
PW-PL-T300	180	120	0.256 (±0.006)	1.684 (±0.021)	15.20 (±0.31)	1.023 (±0.014)
PW-PL-T300	180	140	0.210 (±0.005)	1.686 (±0.025)	12.48 (±0.30)	0.905 (±0.028)
PW-HU-T250	140	100	0.314 (±0.018)	1.758 (±0.047)	17.86 (±0.86)	0.968 (±0.016)
PW-HU-T250	140	120	0.383 (±0.008)	1.596 (±0.013)	23.99 (±0.59)	1.063 (±0.014)
PW-HU-T250	140	140	0.302 (±0.012)	1.675 (±0.009)	18.05 (±0.74)	1.104 (±0.013)
PW-HU-T250	180	100	0.304 (±0.020)	1.820 (±0.014)	16.73 (±1.11)	1.010 (±0.028)
PW-HU-T250	180	120	0.386 (±0.010)	1.639 (±0.013)	23.58 (±0.60)	1.081 (±0.015)
PW-HU-T250	180	140	0.309 (±0.013)	1.742 (±0.011)	17.76 (±0.82)	1.127 (±0.016)
PW-HU-T300	140	100	0.303 (±0.013)	1.254 (±0.019)	24.12 (±0.85)	0.707 (±0.034)
PW-HU-T300	140	120	0.360 (±0.009)	1.462 (±0.029)	24.66 (±0.96)	0.904 (±0.020)
PW-HU-T300	140	140	0.281 (±0.007)	1.268 (±0.013)	22.17 (±0.62)	0.743 (±0.045)
PW-HU-T300	180	100	0.357 (±0.007)	1.503 (±0.037)	23.74 (±0.96)	0.934 (±0.015)
PW-HU-T300	180	120	0.354 (±0.010)	1.544 (±0.012)	22.95 (±0.72)	0.944 (±0.022)
PW-HU-T300	180	140	0.274 (±0.007)	1.361 (±0.014)	20.15 (±0.57)	0.806 (±0.020)
RAW-SW-SK	140	100	0.298 (±0.023)	1.650 (±0.008)	18.09 (±1.41)	1.113 (±0.023)
RAW-SW-SK	140	120	0.315 (±0.011)	1.673 (±0.008)	18.83 (±0.69)	1.183 (±0.013)
RAW-SW-SK	140	140	0.349 (±0.021)	1.709 (±0.014)	20.42 (±1.20)	1.209 (±0.010)
RAW-SW-SK	180	100	0.309 (±0.008)	1.680 (±0.011)	18.38 (±0.45)	1.183 (±0.013)
RAW-SW-SK	180	120	0.327 (±0.015)	1.716 (±0.005)	19.08 (±0.9)	1.176 (±0.010)
RAW-SW-SK	180	140	0.345 (±0.014)	1.756 (±0.010)	19.63 (±0.78)	1.212 (±0.010)
RAW-SW-CZ	140	100	0.206 (±0.006)	1.675 (±0.011)	12.27 (±0.39)	1.107 (±0.016)
RAW-SW-CZ	140	120	0.210 (±0.005)	1.694 (±0.009)	12.41 (±0.27)	1.163 (±0.014)
RAW-SW-CZ	140	140	0.200 (±0.006)	1.723 (±0.012)	11.62 (±0.29)	1.201 (±0.023)
RAW-SW-CZ	180	100	0.209 (±0.007)	1.723 (±0.014)	12.16 (±0.40)	1.147 (±0.023)
RAW-SW-CZ	180	120	0.208 (±0.006)	1.757 (±0.007)	11.86 (±0.33)	1.195 (±0.010)
RAW-SW-CZ	180	140	0.205 (±0.008)	1.792 (±0.015)	11.43 (±0.44)	1.258 (±0.020)
RAW_PW-PL	140	100	0.265 (±0.011)	1.721 (±0.032)	15.38 (±0.49)	1.077 (±0.028)
RAW_PW-PL	140	120	0.248 (±0.030)	1.800 (±0.027)	13.78 (±1.47)	1.183 (±0.023)
RAW_PW-PL	140	140	0.257 (±0.017)	1.812 (±0.026)	14.17 (±0.83)	1.236 (±0.025)
RAW_PW-PL	180	100	0.249 (±0.018)	1.763 (±0.024)	14.12 (±0.86)	1.085 (±0.029)
RAW_PW-PL	180	120	0.243 (±0.018)	1.793 (±0.013)	13.58 (±1.03)	1.170 (±0.018)
RAW_PW-PL	180	140	0.261 (±0.017)	1.872 (±0.022)	13.94 (±0.77)	1.260 (±0.031)
RAW-PW-HU	140	100	0.255 (±0.018)	1.669 (±0.009)	15.29 (±1.13)	1.090 (±0.022)
RAW-PW-HU	140	120	0.272 (±0.010)	1.693 (±0.010)	16.07 (±0.62)	1.140 (±0.016)
RAW-PW-HU	140	140	0.273 (±0.017)	1.744 (±0.020)	15.67 (±0.97)	1.202 (±0.023)
RAW-PW-HU	180	100	0.274 (±0.021)	1.726 (±0.010)	15.87 (±1.31)	1.120 (±0.025)
RAW-PW-HU	180	120	0.267 (±0.022)	1.756 (±0.012)	15.21 (±1.28)	1.187 (±0.014)
RAW-PW-HU	180	140	0.266 (±0.015)	1.798 (±0.008)	14.77 (±0.78)	1.230 (±0.017)

Note: n.a.—not applicable, value not measured

**Table A2:** Basic statistics (means and standard deviations) regarding the physicomechanical properties of pellets produced from the raw and torrefied material.

Material	*P*	*T_a_*	*σ_M_*	*E*	*ε_cM_*	*E_jm_*
	MPa	°C	MPa	MPa	%	J·g^−1^
SW-SK-T250	140	100	1.08 (±0.20)	29.34 (±5.43)	5.62 (±0.74)	0.08 (±0.02)
SW-SK-T250	140	120	1.34 (±0.42)	53.6 (±15.89)	3.77 (±0.77)	0.07 (±0.03)
SW-SK-T250	140	140	1.53 (±0.29)	44.71 (±5.89)	4.90 (±0.85)	0.09 (±0.03)
SW-SK-T250	180	100	1.03 (±0.12)	29.16 (±3.85)	5.43 (±0.39)	0.08 (±0.01)
SW-SK-T250	180	120	1.48 (±0.27)	60.24 (±5.44)	4.12 (±0.90)	0.08 (±0.03)
SW-SK-T250	180	140	2.10 (±0.26)	48.83 (±2.48)	5.94 (±0.29)	0.14 (±0.01)
SW-SK-T300	140	100	0.80 (±0.18)	13.74 (±4.55)	4.90 (±0.53)	0.06 (±0.01)
SW-SK-T300	140	120	1.67 (±0.32)	41.5 (±13.09)	4.57 (±0.45)	0.10 (±0.01)
SW-SK-T300	140	140	1.97 (±0.50)	23.45 (±4.36)	7.60 (±0.72)	0.20 (±0.06)
SW-SK-T300	180	100	1.17 (±0.29)	19.74 (±4.80)	5.58 (±0.63)	0.10 (±0.03)
SW-SK-T300	180	120	1.92 (±0.28)	45.52 (±7.86)	4.81 (±0.34)	0.11 (±0.01)
SW-SK-T300	180	140	1.74 (±0.38)	23.01 (±4.15)	7.30 (±0.59)	0.17 (±0.04)
SW-CZ-T250	140	100	1.70 (±0.23)	27.29 (±3.81)	11.58 (±2.07)	0.31 (±0.06)
SW-CZ-T250	140	120	3.36 (±0.32)	75.63 (±8.50)	6.25 (±0.67)	0.24 (±0.04)
SW-CZ-T250	140	140	4.21 (±0.43)	49.54 (±3.65)	10.12 (±1.37)	0.49 (±0.09)
SW-CZ-T250	180	100	1.83 (±0.26)	33.53 (±4.64)	9.88 (±2.60)	0.26 (±0.07)
SW-CZ-T250	180	120	3.39 (±0.35)	82.92 (±7.95)	5.88 (±0.57)	0.22 (±0.04)
SW-CZ-T250	180	140	4.81 (±0.28)	87.95 (±6.29)	6.10 (±0.54)	0.32 (±0.05)
SW-CZ-T300	140	100	n.a.	n.a.	n.a.	n.a.
SW-CZ-T300	140	120	0.93 (±0.25)	20.12 (±6.72)	6.64 (±1.01)	0.11 (±0.04)
SW-CZ-T300	140	140	0.99 (±0.13)	21.11 (±3.55)	6.15 (±2.48)	0.10 (±0.05)
SW-CZ-T300	180	100	n.a.	n.a.	n.a.	n.a.
SW-CZ-T300	180	120	1.36 (±0.10)	30.86 (±1.73)	6.23 (±0.49)	0.13 (±0.02)
SW-CZ-T300	180	140	1.03 (±0.20)	16.69 (±4.10)	5.64 (±0.68)	0.09 (±0.02)
PW-PL-T250	140	100	2.50 (±0.42)	39.67 (±5.14)	7.60 (±1.53)	0.24 (±0.08)
PW-PL-T250	140	120	3.70 (±0.26)	78.32 (±5.80)	5.32 (±0.70)	0.21 (±0.05)
PW-PL-T250	140	140	4.24 (±0.40)	53.31 (±4.13)	8.06 (±0.95)	0.36 (±0.07)
PW-PL-T250	180	100	2.14 (±0.27)	37.35 (±3.96)	7.47 (±1.79)	0.23 (±0.07)
PW-PL-T250	180	120	3.64 (±0.25)	82.96 (±4.58)	5.50 (±0.98)	0.23 (±0.06)
PW-PL-T250	180	140	4.41 (±0.16)	55.53 (±2.95)	7.50 (±0.79)	0.32 (±0.06)
PW-PL-T300	140	100	1.17 (±0.24)	21.37 (±6.59)	5.84 (±0.67)	0.10 (±0.03)
PW-PL-T300	140	120	3.08 (±0.23)	63.68 (±2.87)	4.83 (±0.32)	0.15 (±0.02)
PW-PL-T300	140	140	1.95 (±0.36)	28.38 (±5.14)	6.65 (±0.50)	0.15 (±0.03)
PW-PL-T300	180	100	1.31 (±0.14)	21.68 (±2.89)	5.74 (±0.38)	0.10 (±0.02)
PW-PL-T300	180	120	3.38 (±0.35)	69.58 (±6.53)	4.34 (±0.36)	0.14 (±0.02)
PW-PL-T300	180	140	2.34 (±0.26)	32.22 (±4.13)	6.92 (±0.46)	0.18 (±0.03)
PW-HU-T250	140	100	2.69 (±0.30)	69.25 (±6.46)	5.04 (±0.51)	0.17 (±0.03)
PW-HU-T250	140	120	2.99 (±0.23)	77.12 (±3.09)	5.20 (±0.44)	0.17 (±0.03)
PW-HU-T250	140	140	4.31 (±0.22)	95.08 (±3.81)	4.03 (±0.30)	0.18 (±0.03)
PW-HU-T250	180	100	2.62 (±0.33)	78.84 (±7.44)	4.73 (±0.66)	0.16 (±0.04)
PW-HU-T250	180	120	3.21 (±0.33)	79.59 (±4.03)	5.35 (±0.82)	0.19 (±0.04)
PW-HU-T250	180	140	4.13 (±0.43)	99.74 (±6.21)	3.75 (±0.44)	0.15 (±0.03)
PW-HU-T300	140	100	0.72 (±0.11)	12.16 (±2.62)	4.77 (±0.64)	0.05 (±0.01)
PW-HU-T300	140	120	1.28 (±0.15)	41.55 (±6.75)	3.60 (±0.85)	0.06 (±0.02)
PW-HU-T300	140	140	1.15 (±0.36)	24.17 (±7.97)	4.28 (±0.62)	0.07 (±0.03)
PW-HU-T300	180	100	1.31 (±0.14)	53.25 (±6.71)	2.78 (±0.56)	0.05 (±0.02)
PW-HU-T300	180	120	1.43 (±0.17)	50.75 (±8.95)	3.44 (±1.07)	0.06 (±0.03)
PW-HU-T300	180	140	1.53 (±0.18)	32.65 (±6.76)	4.01 (±0.29)	0.07 (±0.01)
RAW-SW-SK	140	100	3.83 (±0.35)	80.95 (±5.86)	5.25 (±0.21)	0.23 (±0.02)
RAW-SW-SK	140	120	4.53 (±0.24)	98.48 (±6.73)	5.07 (±0.21)	0.24 (±0.02)
RAW-SW-SK	140	140	5.83 (±0.55)	119.52 (±12.9)	4.42 (±0.16)	0.24 (±0.02)
RAW-SW-SK	180	100	4.53 (±0.24)	98.48 (±6.73)	5.07 (±0.21)	0.24 (±0.02)
RAW-SW-SK	180	120	3.98 (±0.50)	87.85 (±9.39)	4.92 (±0.28)	0.20 (±0.03)
RAW-SW-SK	180	140	5.62 (±0.28)	117.61 (±6.47)	4.46 (±0.21)	0.24 (±0.02)
RAW-SW-CZ	140	100	4.24 (±0.31)	61.70 (±4.31)	8.99 (±1.30)	0.44 (±0.08)
RAW-SW-CZ	140	120	4.46 (±0.23)	73.93 (±5.18)	7.68 (±1.13)	0.38 (±0.08)
RAW-SW-CZ	140	140	5.95 (±0.76)	103.75 (±10.49)	6.71 (±0.78)	0.42 (±0.09)
RAW-SW-CZ	180	100	4.28 (±0.36)	70.26 (±5.95)	7.66 (±0.50)	0.36 (±0.05)
RAW-SW-CZ	180	120	5.23 (±0.37)	88.39 (±6.37)	7.07 (±0.66)	0.38 (±0.05)
RAW-SW-CZ	180	140	6.23 (±0.53)	117.80 (±8.41)	5.43 (±0.29)	0.32 (±0.04)
RAW_PW-PL	140	100	4.64 (±0.41)	90.79 (±7.70)	7.50 (±0.90)	0.45 (±0.08)
RAW_PW-PL	140	120	6.58 (±0.72)	129.87 (±14.67)	6.30 (±0.77)	0.46 (±0.09)
RAW_PW-PL	140	140	8.60 (±0.58)	181.44 (±11.52)	5.20 (±0.50)	0.44 (±0.08)
RAW_PW-PL	180	100	4.67 (±0.56)	86.90 (±8.51)	7.65 (±1.08)	0.45 (±0.10)
RAW_PW-PL	180	120	6.35 (±0.60)	119.10 (±9.88)	8.19 (±3.93)	0.63 (±0.41)
RAW_PW-PL	180	140	8.65 (±0.59)	186.81 (±14.06)	4.88 (±0.59)	0.40 (±0.07)
RAW-PW-HU	140	100	3.60 (±0.22)	66.52 (±5.99)	6.95 (±0.45)	0.29 (±0.03)
RAW-PW-HU	140	120	4.27 (±0.39)	80.44 (±6.04)	5.81 (±0.43)	0.26 (±0.04)
RAW-PW-HU	140	140	4.85 (±0.19)	97.57 (±5.74)	5.02 (±0.30)	0.24 (±0.02)
RAW-PW-HU	180	100	3.82 (±0.25)	77.09 (±5.13)	6.05 (±0.61)	0.26 (±0.04)
RAW-PW-HU	180	120	4.35 (±0.17)	83.55 (±4.38)	5.20 (±0.19)	0.23 (±0.02)
RAW-PW-HU	180	140	5.16 (±0.38)	105.67 (±6.31)	4.70 (±0.31)	0.23 (±0.03)

Note: n.a.—not applicable, value not measured
